# Prevalence and sociodemographic determinants of anemia among ever-married women of reproductive age in Jordan: insights from the 2023 Jordan population and family health survey

**DOI:** 10.1186/s12889-025-22578-7

**Published:** 2025-04-25

**Authors:** Amr Ahmed Aly Ibrahim, Sara Hosny El-Farargy, Rahma Sameh Shaheen, Mahmoud Shaaban Abdelgalil

**Affiliations:** 1 Faculty of Medicine, Menoufia University, Menoufia, Egypt; 2 Faculty of Medicine, Benha University, Benha, Egypt; 3 Faculty of Medicine, Ain-shams University, Cairo, Egypt; 4 Research Insights Arab Network, Cairo, Egypt

**Keywords:** Anemia, Women, Reproductive health, Jordan, Sociodemographic factors

## Abstract

**Background:**

Anemia, characterized by low haemoglobin levels, remains a critical public health issue, particularly among women of reproductive age. Despite global advancements in addressing anemia, it continues to be a widespread concern in Jordan. This study aims to examine the prevalence of anemia and the sociodemographic factors associated with it among ever-married women aged 15–49 in Jordan, utilizing data from the 2023 Jordan Population and Family Health Survey (JPFHS).

**Methods:**

This is a secondary analysis was conducted using data from the 2023 JPFHS. The survey employed a two-stage stratified cluster sampling method to collect comprehensive information on haemoglobin levels, sociodemographic characteristics, and various health-related factors. Anemia was classified into mild, moderate, and severe categories based on WHO guidelines, using hemoglobin levels obtained from capillary blood samples analyzed with portable HemoCue photometers. Multivariate logistic regression analysis was then performed to identify the independent predictors of anemia.

**Results:**

Among the 6,249 participants, the prevalence of anemia was 35.33%, with 1,089 cases of mild anemia, 1,022 moderate cases, and 96 severe cases. The highest incidence of severe and mild anemia was observed in the 45–49 age group (41.1% and 20.2%, respectively). Moderate anemia was most prevalent among women aged 35–39 (21.5%), while the highest proportion of non-anemic participants was found in the 30–34 age group (19.7%). Multivariable analysis showed that women living in northern regions had higher odds of anemia (*P* < 0.0001). Conversely, overweight (*P* = 0.03), obesity (*P* = 0.04), and daily smoking (*P* = 0.002) were associated with lower odds of anemia.

**Conclusion:**

Anemia remains a moderate yet concerning public health issue in Jordan, particularly among underweight women and those in northern regions. The findings emphasize the need for targeted nutritional interventions and region-specific healthcare strategies to address anemia risk. Public health programs should focus on improving dietary intake, especially among underweight women, to mitigate anemia and enhance overall women’s health outcomes.

## Introduction

Anemia is one of the most prevalent health disorders globally, affecting nearly one-third of the world’s population [1]. The World Health Organization (WHO) defines anemia as a condition in which the number of red blood cells or their oxygen-carrying capacity is inadequate to fulfil the body’s physiological requirements [2]. Its pathophysiology is complex, influenced by a range of factors, including nutritional deficiencies, infections, genetic abnormalities, and environmental conditions [1, 3]. Iron deficiency anemia is regarded as the most prevalent nutritional deficiency globally [4]. Anemia is linked to weakened immunity, impaired cognitive function, reduced work capacity, and a diminished overall quality of life [4]. Anemia not only affects individual health but also contributes to broader public health issues, severely reducing quality of life and leading to major complications [5].

Despite a global decline in anemia rates, Jordan has witnessed a steady increase in its prevalence over the past decade, posing significant public health challenges, especially among ever-married women of reproductive age [6, 7]. Women of reproductive age are particularly vulnerable to anemia, with an estimated global prevalence of up to 30% in this group. In this demographic, anemia can impair both maternal and fetal health [8]. Key factors contributing to anemia in women include menstruation, which is the primary risk factor, and pregnancy, where the body may struggle to produce enough red blood cells to meet the increased demands of both the mother and the growing fetus [9, 10]. Around one-third of the older anemic population suffers from deficiencies in folate, iron, and/or vitamin B12. Another third experience chronic inflammation and/or renal insufficiency, while the remaining third have anemia with no clear cause [11].

Recognizing the significant impact of anemia, Jordan has implemented several national surveys, programs, and initiatives to diagnose and address anemia and other micronutrient deficiencies [12, 13]. These efforts include the national wheat flour fortification program, which began in 2001 and incorporates iron and other essential micronutrients. Additionally, the Ministry of Health has introduced routine anemia screenings for pregnant women attending neonatal, prenatal, and postnatal care clinics. Women diagnosed with anemia are provided with supplements containing iron, zinc, and folic acid [12, 13]. Despite these initiatives, however, the prevalence of iron-deficiency anemia has shown only minimal improvement between 2001 and 2019 [14].

The Jordan Population and Family Health Survey (JPFHS) has been a crucial tool for studying anemia and its associated factors among women in Jordan. The 2023 JPFHS reported a high prevalence of anemia among ever-married women of reproductive age- in Jordan, with 32% of women affected. Of these, 17% are classified as mildly anemic, 14% as moderately anemic, and 1% as severely anemic [7]. A prior study by Arabyat et al. analyzed the 2012 JPFHS data and identified several risk factors for anemia, including urban residency, living in the northern or southern regions, having multiple children, being pregnant, and using intrauterine devices (IUDs) as a contraceptive method [15].

With the availability of updated data from the 2023 JPFHS, this study provides an opportunity to reassess previous findings and identify additional sociodemographic factors contributing to anemia. By analyzing the latest data, our research aims to enhance understanding of anemia prevalence and its determinants, offering valuable insights for refining intervention strategies. The findings will inform evidence-based policies and targeted public health programs to reduce anemia prevalence among women in Jordan.

## Methodology

### Study design and data source

This study is a secondary analysis of the 2023 JPFHS, conducted under the Demographic and Health Surveys program. The JPFHS is a nationally representative survey that collects detailed sociodemographic and health-related data across 12 governorates of Jordan. It focuses on ever-married women aged 15–49 years to assess various health indicators, including anemia.

Access to the 2023 JPFHS dataset was obtained through an application to the DHS Program website, which provides publicly available de-identified data for research purposes. Approval for data use was granted upon submission of a formal research proposal outlining the study objectives and methodology.

### Sampling methodology

The JPFHS employed a two-stage stratified cluster sampling method to ensure representativeness across Jordan. In the first stage, primary sampling units (PSUs) were selected based on population size and stratified by urban and rural areas. In the second stage, households were randomly selected within each PSU. This sampling approach ensured a diverse sample reflective of the national population. Further details on the sample design, sampling frame, methodology, and organization of the survey can be found in the final report of the JPFHS 2023 [7].

### Data collection

Data was collected by trained DHS staff using standardized questionnaires and biometric measurements. Hemoglobin levels were obtained through capillary blood samples analyzed using portable HemoCue photometers. This method involves drawing blood from the finger into a reagent-coated microcuvette, which is analysed using a portable, battery-operated photometer to display hemoglobin levels in grams per deciliter. The HemoCue device is a reliable and precise tool for estimating anemia prevalence, yielding results comparable to standard laboratory methods [16]. To ensure accuracy, adjustments were made for altitude and smoking status, as recommended by the WHO [16]. Anemia was categorized into four levels based on hemoglobin concentrations according to the WHO criteria [17]:

- **No anemia**: Hemoglobin ≥ 12.0 g/dL (non-pregnant) or ≥ 11.0 g/dL (pregnant).
- **Mild anemia**: Hemoglobin 11.0–11.9 g/dL (non-pregnant) or 10.0–10.9 g/dL (pregnant).
- **Moderate anemia**: Hemoglobin 8.0–10.9 g/dL (non-pregnant) or or Hemoglobin 7.0–9.9 g/dL (pregnant).
- **Severe anemia**: Hemoglobin < 8.0 g/dL(non-pregnant) or Hemoglobin > 7 g/dL (pregnant).

### Inclusion and exclusion criteria

This study included ever-married women of reproductive age (15–49 years) who participated in the 2023 JPFHS and had available hemoglobin measurements. Women with missing anemia data were excluded to maintain the accuracy and completeness of the analysis.

### Study variables

The primary outcome was anemia status. Independent variables analysed included:

- **Sociodemographic Factors**: Age groups, educational attainment, wealth index, marital status, urban/rural residence, and geographic region.
- **Health-Related Factors**: Parity, body mass index (BMI), breastfeeding status, smoking habits, and health insurance coverage.

Household economic status was assessed using the wealth index, derived from data on household assets. This index classified households into five wealth quintiles: poorest, poor, middle, rich, and richest [18]. BMI was used to assess the overall nutritional status of women in the survey. BMI is calculated by dividing weight in kilograms by height in meters squared (kg/m²). Women were categorized as underweight (< 18.5 kg/m²), normal weight (18.5–24.9 kg/m²), overweight (25–29.9 kg/m²) or obese (< 30 kg/m²) [19].

### Statistical analysis

Data analysis was performed using SPSS version 24, with a weighted count based on DHS recommendations [20]. The sample weight, an eight-digit variable with six implied decimal places, was divided by 1,000,000 for application. Descriptive statistics were used to summarize the study population’s characteristics, presenting results as frequencies and percentages. A multiple logistic regression model was constructed, treating anemia level as a binary variable, comparing individuals with no anemia to those with any form of anemia (mild, moderate, or severe). The choice of using individuals with no anemia as the reference group aligns with standard epidemiological practices in logistic regression. This approach facilitates a clear comparison between those affected by anemia (regardless of severity) and those without, allowing for a more straightforward interpretation of risk factors associated with anemia. The results were expressed as adjusted odds ratios (AORs) with 95% confidence intervals (CIs). Statistical significance was set at a p-value of less than 0.05.

### Ethical considerations

Ethical approval for the JPFHS was obtained by the DHS program before data collection. Participants provided informed consent at the time of the survey. This secondary analysis used anonymized data obtained from the DHS Program after approval of a research proposal. No additional ethical approval was required as the analysis involved de-identified data.

## Results

### Baseline characteristics

A total of 6249 women were included in this study (4042 Non anemic, 1089 Mild anemia, 1022 Moderate, and 96 Severe anemia) from the JPFHS 2023. Among the age groups, the 45–49 group shows the highest incidence of severe and mild anemia 41.1%, and 20.2%, respectively. While the 35–39 group shows a high incidence of moderate anemia 21.5% and the 30–34 group shows a high incidence of non-anemic participants 19.7%.

The distribution of BMI among women in the study population varied across different anemia levels. Among those classified as underweight, the prevalence of anemia was relatively low, with only a small percentage of women in each anemia category (severe: 0.3%, moderate: 2.0%, mild: 2.0%, and not anemic: 1.3%). In contrast, women with a normal weight exhibited a higher proportion of anemia cases, particularly in the moderate and mild categories, accounting for 26.4% and 26.8%, respectively, in the severe and moderate anemia groups. Women who were overweight had a notable representation across all anemia levels, with 21.5% experiencing severe anemia, 34.4% moderate anemia, and 34.3% mild anemia. The highest percentage of anemia was observed in the obese group, where 51.8% of women had severe anemia, 36.7% had moderate anemia, 37.8% had mild anemia, and 35.9% were not anemic.

Anemia prevalence varied by region in Jordan. In the central region, the majority of women had severe (63.6%) and moderate anemia (60.0%), with 72.5% not anemic. The northern region had lower anemia rates, with 33.1% severe, 33.2% moderate, and 21.7% not anemic. The southern region had the lowest anemia rates, with 3.3% severe, 6.8% moderate, and 5.8% not anemic. Most participants were from urban areas.

Educationally, Women with no education had the lowest rates of severe (0.4%) and moderate (1.9%) anemia. Those with primary education had higher rates, with 13.6% severely anemic and 8.4% moderately anemic. The majority of women had secondary education, with 64.5% severely anemic and 61.6% moderately anemic. Women with higher education had the lowest anemia rates.More details about the baseline characteristics of the participants categorized according to anemia level are reported in Table 1.

**Table 1:** Baseline characteristics of the participants

Variables		Anemia level			
		Severe	Moderate	Mild	Not anemic
		N (%)	N (%)	N (%)	N (%)
**Age in 5-year groups**	**15–19**	0 (0.0%)	21 (2.1%)	16 (1.5%)	74 (1.8%)
	**20–24**	1 (1.0%)	54 (5.3%)	105 (9.7%)	333 (8.3%)
	**25–29**	11 (11.4%)	139 (13.6%)	180 (16.5%)	615 (15.2%)
	**30–34**	12 (12.3%)	169 (16.5%)	186 (17.1%)	799 (19.8%)
	**35–39**	16 (16.8%)	220 (21.5%)	209 (19.2%)	727 (18.0%)
	**40–44**	17 (17.4%)	217 (21.2%)	173 (15.9%)	699 (17.3%)
	**45–49**	40 (41.1%)	203 (19.8%)	220 (20.2%)	794 (19.7%)
**BMI**	**Underweight**	0 (0.3%)	21 (2.0%)	21 (2.0%)	54 (1.3%)
	**Normal weight**	25 (26.4%)	274 (26.8%)	283 (26.0%)	1096 (27.1%)
	**Overweight**	21 (21.5%)	351 (34.4%)	374 (34.3%)	1439 (35.6%)
	**Obese**	50 (51.8%)	376 (36.7%)	411 (37.8%)	1452 (35.9%)
**Region**	**Central**	61 (63.6%)	614 (60.0%)	610 (56.0%)	2931 (72.5%)
	**North**	32 (33.1%)	339 (33.2%)	406 (37.3%)	878 (21.7%)
	**South**	3 (3.3%)	69 (6.8%)	73 (6.7%)	232 (5.8%)
**Type of place of residence**	**Urban**	81 (83.7%)	915 (89.5%)	964 (88.5%)	3732 (92.3%)
	**Rural**	16 (16.3%)	107 (10.5%)	125 (11.5%)	310 (7.7%)
**Highest educational level**	**No education**	0 (0.4%)	20 (1.9%)	32 (2.9%)	78 (1.9%)
	**Primary**	13 (13.6%)	86 (8.4%)	89 (8.2%)	275 (6.8%)
	**Secondary**	62 (64.5%)	630 (61.6%)	612 (56.2%)	2261 (56.0%)
	**Higher**	21 (21.6%)	287 (28.0%)	356 (32.7%)	1427 (35.3%)
**Wealth index combined**	**Poorest**	20 (20.9%)	276 (27.0%)	250 (23.0%)	798 (19.7%)
	**Poorer**	29 (30.5%)	235 (23.0%)	233 (21.4%)	850 (21.0%)
	**Middle**	26 (26.6%)	246 (24.0%)	244 (22.4%)	836 (20.7%)
	**Richer**	20 (20.9%)	158 (15.5%)	198 (18.1%)	781 (19.3%)
	**Richest**	1 (1.1%)	107 (10.5%)	165 (15.1%)	777 (19.2%)
**Frequency smokes cigarettes**	**Does not smoke**	89 (92.3%)	974 (95.3%)	1015 (93.2%)	3630 (89.8%)
	**Every day**	3 (3.5%)	40 (3.9%)	53 (4.8%)	326 (8.1%)
	**Some days**	4 (4.2%)	8 (0.8%)	22 (2.0%)	86 (2.1%)
**Current marital status**	**Never in union**	0 (0.0%)	0 (0.0%)	0 (0.0%)	0 (0.0%)
	**Married**	86 (88.9%)	944 (92.3%)	1022 (93.8%)	3723 (92.1%)
	**Living with partner**	0 (0.0%)	0 (0.0%)	0 (0.0%)	0 (0.0%)
	**Widowed**	9 (9.2%)	34 (3.4%)	14 (1.3%)	107 (2.6%)
	**Divorced**	2 (1.9%)	41 (4.0%)	53 (4.9%)	209 (5.2%)
	**No longer living together/separated**	0 (0.0%)	3 (0.3%)	0 (0.0%)	3 (0.1%)
**Currently pregnant**	**No or unsure**	96 (99.7%)	966 (94.5%)	1006 (92.3%)	3743 (92.6%)
	**Yes**	0 (0.3%)	56 (5.5%)	84 (7.7%)	298 (7.4%)
**Parity**	**Nulliparous**	3 (3.2%)	49 (4.8%)	72 (6.6%)	325 (8.1%)
	**Primiparous**	5 (5.6%)	82 (8.0%)	128 (11.7%)	452 (11.2%)
	**Multiparous**	88 (91.3%)	890 (87.1%)	890 (81.7%)	3264 (80.8%)
**Currently breastfeeding**	**No**	94 (97.5%)	917 (89.7%)	977 (89.7%)	3574 (88.4%)
	**Yes**	2 (2.5%)	105 (10.3%)	112 (10.3%)	468 (11.6%)
**The method used last sexual intercourse: IUD**	**No**	39 (88.6%)	409 (71.1%)	435 (68.9%)	1549 (68.4%)
	**Yes**	5 (11.4%)	166 (28.9%)	196 (31.1%)	714 (31.6%)
**Respondent currently working**	**No**	93 (96.9%)	930 (91.0%)	964 (88.5%)	3502 (86.6%)
	**Yes**	3 (3.1%)	92 (9.0%)	125 (11.5%)	540 (13.4%)
**Covered by health insurance**	**No**	21 (22.0%)	289 (28.3%)	312 (28.7%)	1344 (33.3%)
	**Yes**	75 (78.0%)	733 (71.7%)	777 (71.3%)	2698 (66.7%)

BMI: Body mass index, IUD: Intrauterine Device, N: Number

### Regression analysis of factors associated with anaemia among ever-married women aged 15–49 in Jordan

The results of the multivariate logistic regression showed significant associations between BMI and anemia. Compared to underweight women, normal-weight women had a slightly lower odds of anemia, but this association was not statistically significant (AOR = 0.457, 95% CI: 0.201–1.039, *p* = 0.062). Overweight women had a significantly lower odds of anemia (AOR = 0.413, 95% CI: 0.186–0.919, *p* = 0.030), as did obese women (AOR = 0.419, 95% CI: 0.185–0.953, *p* = 0.038).

Regarding frequency of smoking, women who smoked every day had significantly lower odds of experiencing anemia compared to non-smokers (AOR = 0.401, 95% CI: 0.224–0.719, *p* = 0.002). On the other hand, women who smoked on some days did not demonstrate a statistically significant difference in anemia risk (AOR = 0.378, 95% CI: 0.123–1.160, *p* = 0.089).

Regarding region, our analysis revealed significant regional differences in anemia risk. Women from the northern region had significantly higher odds of anemia compared to those in the central region (AOR = 1.768, 95% CI: 1.390–2.250, *p* < 0.001). In contrast, women from the southern region did not show a significant difference in anemia risk compared to those from the central region (AOR = 1.168, 95% CI: 0.913–1.493, *p* = 0.217).

Our analysis found no significant association between anemia and various sociodemographic factors, including parity status, health insurance coverage, employment status, use of IUD, breastfeeding, current marital status, wealth index, highest educational level, type of place of residence, and age in 5-year groups.

More details about multivariate logistic regression analysis are reported in Table 2.

**Table 2:** Regression Analysis of Factors Associated with Anaemia among ever-married women aged 15–49, Jordan

Parameter Estimates							
Variables		B	Std. Error	AOR	95% Confidence Interval for AOR		*P*. Value
					Lower	Upper	
**Age in 5-year groups**	**15–19**	**Reference**					
	**20–24**	-0.91	0.692	0.403	0.103	1.567	0.189
	**25–29**	-0.342	0.687	0.71	0.184	2.736	0.619
	**30–34**	-0.48	0.705	0.619	0.155	2.469	0.496
	**35–39**	-0.12	0.723	0.887	0.215	3.664	0.868
	**40–44**	-0.206	0.706	0.814	0.204	3.252	0.77
	**45–49**	-0.441	0.714	0.643	0.159	2.61	0.537
**Wealth index combined**	**Poorest**	**Reference**					
	**Poorer**	-0.088	0.157	0.915	0.673	1.245	0.573
	**Middle**	0.123	0.175	1.13	0.802	1.593	0.483
	**Richer**	-0.141	0.19	0.868	0.597	1.262	0.458
	**Richest**	-0.431	0.242	0.65	0.405	1.045	0.075
**Highest educational level**	**No education**	**Reference**					
	**Primary**	0.429	0.454	1.536	0.63	3.743	0.345
	**Secondary**	0.04	0.408	1.041	0.468	2.317	0.921
	**Higher**	-0.099	0.419	0.906	0.398	2.06	0.813
**Type of place of residence**	**Urban**	**Reference**					
	**Rural**	0.174	0.136	1.19	0.911	1.554	0.201
**Region**	**Central**	**Reference**					
	**North**	0.57	0.123	1.768	1.39	2.25	**> 0.0001**
	**South**	0.155	0.125	1.168	0.913	1.493	0.217
**Parity status**	**Nulliparity**	**Reference**					
	**Primiparity**	0.165	0.852	1.18	0.222	6.278	0.846
	**multiparity**	0.412	0.83	1.51	0.296	7.694	0.619
**BMI**	**Underweight**	**Reference**					
	**Normal weight**	-0.784	0.419	0.457	0.201	1.039	0.062
	**Overweight**	-0.884	0.408	0.413	0.186	0.919	**0.03**
	**Obese**	-0.869	0.418	0.419	0.185	0.953	**0.038**
**Covered by health insurance**	**No**	**Reference**					
	**Yes**	0.018	0.15	1.019	0.759	1.366	0.902
**Respondent currently working**	**No**	**Reference**					
	**Yes**	-0.05	0.194	0.952	0.65	1.392	0.798
**Using IUD**	**No**	**Reference**					
	**Yes**	-0.129	0.134	0.879	0.676	1.143	0.336
**Currently breastfeeding**	**No**	**Reference**					
	**Yes**	-0.163	0.179	0.849	0.598	1.207	0.362
**Current marital status**	**Married**	**Reference**					
	**Widowed**	0.276	1.193	1.318	0.127	13.697	0.817
	**Divorced**	0.213	1.248	1.238	0.107	14.331	0.864
	**No longer living together/separated**	0.719	1.053	2.052	0.26	16.193	0.495
**Frequency of smoking**	**Does not smoke**	**Reference**					
	**Every day**	-0.914	0.298	0.401	0.224	0.719	**0.002**
	**Some days**	-0.973	0.571	0.378	0.123	1.16	0.089

B: β coefficient, Std. Error: Standard error, AOR: Adjusted odds ratio, BMI: Body mass index, IUD: Intrauterine Device

## Discussion

### Key findings

This study offers a detailed analysis of anemia prevalence and its contributing factors among ever-married women aged 15–49 years in Jordan, utilizing data from the 2023 JPFHS. The overall anemia prevalence was 35.33%, which is lower than the 37% reported in the 2012 JPFHS [15] and the 43% reported in the 2017/2018 JPFHS [21]. Additionally, it is 8% below the World Health Organization’s threshold of 40%, which classifies anemia as a “severe public health problem” [22].

Our analysis revealed a significant association between BMI and anemia, with overweight and obese participants showing a lower risk of anemia compared to those who were underweight. These findings align with studies by Harding et al. (2017) in Nepal and Pakistan [23] and Gebremedhin et al. (2005) in Ethiopia [24]. However, they contrast with the results of Arabyat et al. (2019) in Jordan [15], which found no significant association between BMI and anemia. Obesity has a complex and sometimes contradictory relationship with iron status in women of reproductive age (WRA), with the existing research showing a variety of associations. A systematic review by Rachmah et al. (2024) examined the relationship between overweight/obesity and iron-deficiency anemia (IDA) among women of reproductive age (WRA), analyzing findings from 27 studies [25]. The review found that most studies reported no significant association between overweight/obesity and hemoglobin levels overall. However, a positive association was more commonly observed in pregnant women [25].

The link between overweight/obesity and serum ferritin levels varied. For non-pregnant women, most studies indicated a positive association, while fewer studies explored hepcidin and inflammatory markers. Among these, the majority reported elevated levels in overweight/obese WRA [25].

Among pregnant women, overweight/obesity was positively associated with anemia and IDA but showed a negative association with iron deficiency (ID). This could be due to co-morbidities like sleep apnea that cause polycythemia, or potentially higher iron intake. In contrast, overweight/obese non-pregnant women were positively linked to anemia, ID, and IDA [25].

Obesity is associated with low-grade chronic inflammation, which increases hepcidin synthesis—a hormone that regulates iron absorption and availability. Elevated hepcidin levels can impair iron absorption and bioavailability, potentially contributing to iron deficiency [25]. Additionally, while serum ferritin is a marker of iron stores, it also functions as an acute-phase reactant, meaning higher ferritin levels in overweight/obese women may reflect inflammation rather than increased iron stores [25]. The systematic review found that some studies have reported lower transferrin saturation and decreased serum iron levels among overweight/obese women, likely due to impaired iron absorption or inflammation-related factors [25]. Despite these challenges, our finding of lower anemia risk among overweight and obese women could be linked to factors such as higher overall nutritional intake or differences in metabolic adaptations, highlighting the need for further research to fully understand these dynamics.

Our analysis found that smoking was associated with lower odds of anemia, particularly among daily smokers. This finding contrasts with the study by Arabyat et al. (2019) in Jordan [15], which reported no association between smoking and anemia. A key difference is that Arabyat et al. [15] classified smoking as a binary variable (smoker vs. non-smoker), whereas our study examined smoking frequency. Our results suggest that merely being a smoker is not enough to influence anemia risk; rather, the inverse association becomes more pronounced with higher smoking frequency. This is further supported by our observation that occasional smoking (“some days”) showed no significant association with anemia.

Cigarette smoke contains carbon monoxide, which binds to hemoglobin with a much higher affinity than oxygen, forming carboxyhemoglobin. This reduces the oxygen-carrying capacity of the blood, leading to tissue hypoxia [26]. In response, the body may stimulate red blood cell production to compensate for reduced oxygen delivery, resulting in higher hemoglobin levels among smokers. This physiological adaptation can obscure anemia detection, as elevated hemoglobin levels may mask underlying iron deficiencies [26]. Studies have shown that smokers generally have higher hemoglobin concentrations than non-smokers [26–28]. To account for this effect, adjusted hemoglobin cutoff values are used for smokers to improve the accuracy of anemia diagnosis [16]. However, it is important to emphasize that while smoking is associated with higher hemoglobin levels, this does not indicate a health benefit. Smoking is linked to numerous adverse health outcomes, including an increased risk of cardiovascular disease, chronic obstructive pulmonary disease (COPD), lung cancer, and stroke [29–32]. Additionally, smoking-induced increases in hemoglobin levels can contribute to increased blood viscosity, which may elevate the risk of thrombosis and other vascular complications [30]. The rise in hemoglobin levels among smokers is not due to improved hematologic health but rather a compensatory response to chronic hypoxia caused by carbon monoxide exposure [26]. This adaptation does not address underlying iron deficiencies or improve overall oxygen delivery efficiency. Therefore, the increase in hemoglobin associated with smoking should not be interpreted as beneficial, as it occurs at the expense of significant harm to overall health [30].

Our analysis revealed a significant association between geographic regions and anemia risk. Individuals residing in the northern region were found to have a higher risk of anemia compared to those in the central region. In contrast, no significant association with anemia was observed for individuals living in the southern region. This finding aligns with Abdo et al. (2019) in Jordan [33], while it contrasts with the results of Arabyat et al. (2019) in Jordan [15], which showed that individuals in the northern and southern regions of Jordan had a higher risk of anemia in 2012. The discrepancy between our findings and those of Arabyat et al. [15] may reflect improvements in the southern region over the past decade, possibly due to factors such as better healthcare access, nutritional interventions, or socioeconomic changes that have contributed to reducing anemia prevalence.

The higher anemia prevalence in the northern region may be attributed to several factors, including lower socioeconomic status and geographical characteristics, such as living at low altitudes below sea level. Research has shown that hemoglobin levels are often lower at lower altitudes. A 2012 study in Jordan, for instance, found that individuals living below sea level had significantly lower hemoglobin levels compared to those residing above sea level [34]. Additionally, the northern region’s rural nature and limited access to resources may contribute to poorer nutritional status, which is a key determinant of anemia. Studies have consistently shown that rural residents are at a much higher risk of developing anemia than those in urban areas, with a more than two-fold increased risk [35, 36]. To better understand the factors driving anemia prevalence, further research should explore the complex interactions between geographic, socioeconomic, and environmental variables.

Our analysis revealed no significant link between anemia and women’s education or wealth, differing from previous research conducted in Tanzania [37], Mali [38], and India [39], which found that women with lower education and income levels faced a higher risk of anemia. Anemia is often linked to disadvantaged groups, as less educated and lower-income women typically have limited access to health information and healthcare services compared to their more educated and wealthier peers. However, our analysis showed no variation in health insurance coverage between anemic and non-anemic women, which could explain the absence of disparities in anemia prevalence based on education and wealth in our findings.

Our analysis found no significant association between age and anemia. This contrasts with previous studies that indicated older ever-married women are at a higher risk of anemia compared to their younger counterparts [11, 40, 41]. Factors such as poor dietary habits and the presence of comorbidities are likely contributors to increased susceptibility to anemia among older adults [42]. Additionally, recent clinical data suggest that anemia is often underdiagnosed and undertreated in elderly populations, partly due to the misconception that anemia is a natural consequence of aging [43]. Proper evaluation of anemia in older adults is essential to identify its underlying causes and ensure appropriate treatment in accordance with established guidelines for the diagnosis and management of anemia in this age group [44, 45].

Our analysis found no significant association between IUD use and anemia, which contrasts with the findings of Arabyat et al. (2019) in Jordan [15], where IUD use was associated with a significant increase in anemia risk. The use of copper IUDs as a contraceptive method may elevate the risk of iron-deficiency anemia due to increased menstrual blood loss [15]. However, the JPFHS data does not include information on the specific type of IUD used, limiting our ability to clarify the differences observed between the two studies.

Research on the association between IUD use and anemia presents mixed findings. A hospital-based study in Egypt reported significantly lower hemoglobin levels in IUD users compared to non-users or users of other contraceptive methods [46]. Conversely, a longitudinal study in Iran found no significant increase in anemia cases among copper-IUD users [47]. Meta-analyses also revealed slight decreases in hemoglobin levels after one year of copper-IUD use, but these changes were insufficient to cause anemia in women without a prior history of the condition [48]. Similarly, another systematic review found no significant hemoglobin changes in anemic women using copper IUDs for up to a year [49].

Lastly, our study did not find a significant association between parity or breastfeeding and anemia, which contrasts with findings from Arabyat et al. (2019) in Jordan [15], where higher parity was linked to increased anemia risk, and Harding et al. (2017) in Nepal [23], which reported a relationship between breastfeeding and anemia. This discrepancy may be attributed to improvements in maternal care during the antepartum and postpartum periods, including better nutritional support and iron supplementation, which mitigate the risk factors associated with high parity and extended breastfeeding.

High parity has traditionally been associated with increased anemia risk due to factors such as frequent episodes of hemorrhage before, during, or after delivery, and the depletion of micronutrients, including iron, from repeated pregnancies and breastfeeding. Inadequate replenishment of these nutrients can elevate anemia risk in multiparous women [50, 51].

### Strengths and limitations

The key strengths of this study lie in its use of a nationally representative sample of ever-married women in Jordan aged 15–49 years, enabling the results to be broadly applicable. Furthermore, the study directly measured hemoglobin levels, eliminating the reliance on medical records or self-reported data. Additionally, the application of multivariate regression analysis allowed for a comprehensive examination of the risk factors associated with anemia.

A key limitation of this study is its reliance on cross-sectional data, which limits the ability to infer causal relationships between the risk factors and anemia. Although the study used a nationally representative sample of ever-married women aged 15–49 years, the results may not be fully applicable to unmarried women or individuals outside this age group, which may reduce the generalizability. Another limitation is the lack of detailed information on the specific types of IUDs used by participants, which could have influenced the observed association between IUD use and anemia. Additionally, although BMI was used as an indicator of nutritional status, no significant relationship with anemia was found, and BMI alone is not a fully accurate measure of nutritional status because it does not differentiate between fat and lean body mass. The study also relied heavily on self-reported data, which could have introduced recall or social desirability biases, potentially affecting the reliability of the findings. Lastly, the study did not gather detailed information on participants’ dietary habits or any chronic health conditions, which could have acted as confounding factors influencing anemia prevalence.

### Recommendation

Given the identified associations between anemia and BMI, smoking status, and regional disparities, targeted public health strategies are essential. Nutrition-focused interventions should prioritize underweight women, ensuring adequate iron intake and improving dietary diversity to reduce anemia risk. While overweight and obese women showed lower odds of anemia, their overall health risks necessitate balanced dietary and lifestyle interventions.

The significant regional differences in anemia prevalence, particularly the higher odds among women in the northern region, highlight the need for region-specific public health policies. Efforts should focus on improving healthcare access, enhancing nutritional education, and ensuring equitable distribution of iron supplementation programs.The inverse association between daily smoking and anemia risk, while statistically significant, does not support smoking as a preventive measure.

Finally, the lack of significant associations with key sociodemographic factors suggests that anemia prevention programs should adopt a broader, population-wide approach, incorporating regular screenings, community-based nutrition programs, and targeted awareness campaigns to improve iron intake and overall health outcomes.

## Conclusion

Women residing in the northern region had higher odds of anemia, whereas overweight, obesity and daily smoking were associated with lower odds. These findings emphasize the need for targeted interventions to address anemia among women in northern regions and those with lower BMI, aiming to reduce anemia prevalence and improve overall women’s health.

## Data Availability

Data is available upon request from ICF International’s website (https://dhsprogram.com/data/available-datasets.cfm).
